# Ferroptosis and myocardial ischemia-reperfusion: mechanistic insights and new therapeutic perspectives

**DOI:** 10.3389/fphar.2024.1482986

**Published:** 2024-10-01

**Authors:** Binwei Jin, Zhiming Zhang, Yang Zhang, Minjun Yang, Cheng Wang, Jiayi Xu, Yu Zhu, Yafei Mi, Jianjun Jiang, Zhenzhu Sun

**Affiliations:** ^1^ Department of Cardiology, Taizhou Hospital of Zhejiang Province Affiliated to Wenzhou Medical University, Linhai, China; ^2^ Laboratory of Cardiovascular Disease, Taizhou Hospital of Zhejiang Province Affiliated to Wenzhou Medical University, Linhai, China; ^3^ Department of Cardiology, Taizhou hospital of Zhejiang Province, Shaoxing University, Linhai, China; ^4^ Medical Research Center, Taizhou Hospital of Zhejiang Province, Linhai, China

**Keywords:** ferroptosis, myocardial ischemia-reperfusion injury, reactive oxygen species, iron metabolism, lipid peroxidation, therapeutic strategies

## Abstract

Myocardial ischemia-reperfusion injury (MIRI) is a significant factor in the development of cardiac dysfunction following a myocardial infarction. Ferroptosis, a type of regulated cell death driven by iron and marked by lipid peroxidation, has garnered growing interest for its crucial involvement in the pathogenesis of MIRI.This review comprehensively examines the mechanisms of ferroptosis, focusing on its regulation through iron metabolism, lipid peroxidation, VDAC signaling, and antioxidant system dysregulation. We also compare ferroptosis with other forms of cell death to highlight its distinct characteristics. Furthermore, the involvement of ferroptosis in MIRI is examined with a focus on recent discoveries concerning ROS generation, mitochondrial impairment, autophagic processes, ER stress, and non-coding RNA regulation. Lastly, emerging therapeutic strategies that inhibit ferroptosis to mitigate MIRI are reviewed, providing new insights into potential clinical applications.

## 1 Introduction

MIRI is a significant pathological event that occurs after the restoration of blood flow to ischemic myocardial tissue following an acute myocardial infarction (AMI). Although timely reperfusion is critical to salvage myocardial tissue and restore organ function, it paradoxically leads to further myocardial injury, exacerbating the initial ischemic damage. This paradoxical injury known as MIRI initiates a series of pathophysiological responses, such as oxidative damage, inflammatory processes, mitochondrial disruption, calcium imbalance, and endothelial injury, all leading to cardiomyocyte death and compromised cardiac function (Hausenloy and Yellon, 2013; Ibáñez et al., 2015; Heusch, 2020; Jennings, 2013). Different types of cellular death, such as necrosis, apoptosis, autophagy, and pyroptosis, have been associated with MIRI, underscoring the intricate nature of this condition.

While necrosis and apoptosis have been widely explored in the context of MIRI, recent studies are increasingly concentrating on ferroptosis, a newly identified and unique type of regulated cell death. Ferroptosis is particularly noteworthy due to its unique iron-dependent nature and the involvement of lipid peroxidation, setting it apart from other cell death mechanisms.Unlike apoptosis, which involves nuclear fragmentation and DNA degradation, ferroptosis is defined by the shrinkage of mitochondria, an increase in membrane density, and the accumulation of non-heme iron and lipid ROS (Dixon et al., 2012a; Baba et al., 2018; Yang T. et al., 2023). These distinct features have led to growing attention on ferroptosis in MIRI, especially because it plays a critical role in exacerbating myocardial injury during reperfusion, when oxidative stress is at its peak.

What makes ferroptosis particularly compelling in the context of MIRI is its resistance to traditional cell death inhibitors, which are often effective against apoptosis or necrosis. This resistance arises from ferroptosis’ dependence on iron metabolism and lipid peroxidation pathways, which are not targeted by conventional cell death pathways (Yang T. et al., 2023; Cai et al., 2023; Feng et al., 2019). As a result, ferroptosis represents a new therapeutic target in the treatment of MIRI, especially considering that traditional methods of managing reperfusion injury have not been fully successful in mitigating cardiomyocyte death (Yang T. et al., 2023; Gao et al., 2015; Williams et al., 1991).

Moreover, ferroptosis is closely linked to the oxidative stress that occurs during MIRI. The reperfusion phase, marked by the reintroduction of oxygen, leads to a burst of ROS, which triggers ferroptosis through lipid peroxidation. In addition, ischemia-induced iron accumulation creates a pro-ferroptotic environment, further amplifying ROS production through the Fenton reaction (Ma et al., 2022; Moon et al., 2020). This makes ferroptosis highly relevant to MIRI, as it not only contributes to cell death but also worsens oxidative damage through a self-perpetuating cycle of iron overload and ROS accumulation.

Additionally, ferroptosis is especially significant due to its connection with GPX4, an enzyme crucial for safeguarding cells against lipid peroxidation. In MIRI, GPX4 levels are often depleted, leading to increased susceptibility to ferroptosis (Feng et al., 2019; Gao et al., 2024; Lu et al., 2022). This depletion of GPX4, combined with the accumulation of iron and ROS, further underscores why ferroptosis plays a key role in MIRI. Targeting ferroptosis by restoring GPX4 activity or using iron chelators has shown promise in reducing myocardial injury, making it a novel therapeutic approach for managing MIRI (Gao et al., 2024; Lu et al., 2022).

Despite these advancements, the detailed molecular pathways of ferroptosis in MIRI are still not fully elucidated.Current evidence indicates that irregular iron homeostasis, altered amino acid pathways, and heightened lipid peroxidation are the main contributors to ferroptosis in cardiomyocytes (Cai et al., 2023; Feng et al., 2019; Miyamoto et al., 2022).Further exploration of these pathways could unlock new therapeutic targets, offering more comprehensive protection against reperfusion injury and improving clinical outcomes for patients.

This review seeks to thoroughly examine the involvement of ferroptosis in MIRI, with an emphasis on new therapeutic approaches targeting ferroptosis-associated pathways.By consolidating existing insights into the molecular mechanisms underlying ferroptosis and assessing innovative therapeutic strategies, this review aims to provide fresh perspectives on managing MIRI and to underscore promising avenues for future research.

## 2 Ferroptosis

### 2.1 Overview

Ferroptosis is defined as a type of programmed cell death that is dependent on iron and distinct from apoptosis, a concept first proposed by Dixon and his team in 2012 (Dixon et al., 2012b). This mechanism involves the accumulation of iron-dependent ROS that exceed the cell’s capacity to maintain redox balance, leading to lipid peroxidation and, ultimately, cell death. The primary morphological features of ferroptosis include the contraction of mitochondria, increased density of the mitochondrial membrane, and the disruption of mitochondrial cristae, while the morphology of the cell nucleus remains unchanged. Studies have demonstrated that compounds like erastin and RSL3 can trigger ferroptosis in cells. Iron chelators and antioxidants can prevent these types of cell death (Chen Y. et al., 2023; Park et al., 2023). Among these, GPX-4 serves as a vital regulatory component in the process of ferroptosis (Han et al., 2021; Krümmel et al., 2021; Yang et al., 2016).

**FIGURE 1:**
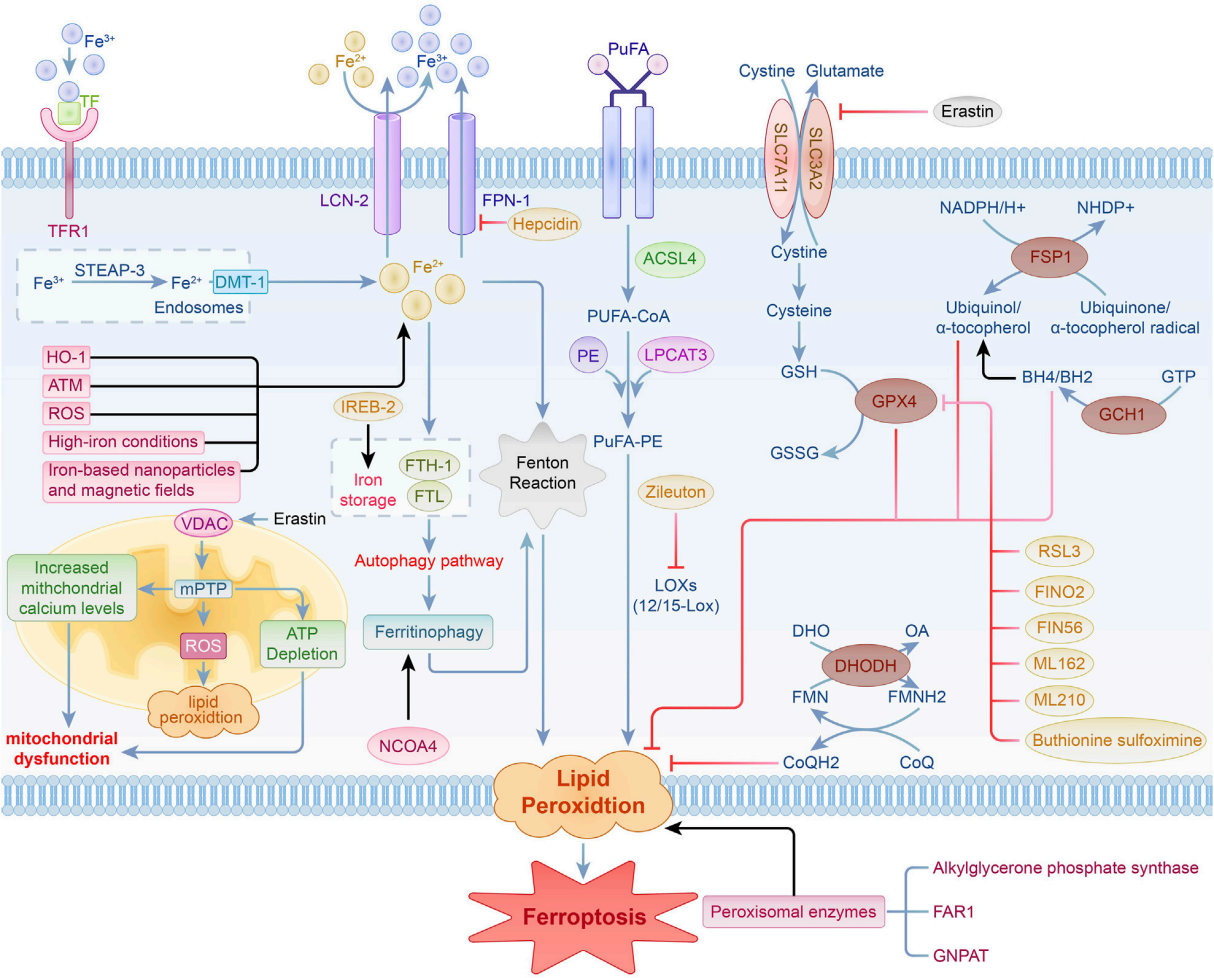
Regulation of iron metabolism, lipid peroxidation, the VDAC signaling pathway, and antioxidant system pathways in ferroptosis.

The mechanisms underlying ferroptosis are intricate, encompassing the buildup of lipid peroxides, an increase in divalent iron ions (Fe^2^⁺), mitochondrial-dependent pathways, and related metabolic signaling pathways. Ongoing research has provided a preliminary understanding of the ferroptosis process, revealing that it is primarily regulated by various intracellular signaling pathways. These mechanisms include the modulation of iron metabolism, control of lipid peroxidation, antioxidant systems such as the Nrf2 signaling pathway, and the voltage-dependent anion channel (VDAC) pathway (Rochette et al., 2023; Zhang et al., 2023; Lv et al., 2023a; Rochette et al., 2022; Sun Y. et al., 2022) (Figure 1). These studies establish a foundation for exploring the involvement of ferroptosis in diverse diseases and its possible therapeutic strategies.

### 2.2 Molecular mechanisms

#### 2.2.1 Regulation of iron metabolism

Iron is an essential trace element crucial for cell vitality, contributing significantly to various biological functions, including electron transfer, respiration, DNA synthesis, cellular growth and differentiation, as well as gene expression regulation. The regulation and preservation of iron homeostasis are intricate processes. Excessive iron ion accumulation can elevate reactive oxygen species (ROS) production via the Fenton reaction, resulting in significant organ damage (Dixon et al., 2012a; Rafati Rahimzadeh et al., 2023).

In iron metabolism, iron is found in the forms of Fe^2^⁺ and Fe³⁺. Ferric iron (Fe³⁺) attaches to circulating transferrin (TF) and is transported into cells via the transferrin receptor 1 (TFR-1), where it is localized in endosomes. Inside endosomes, Fe³⁺ is converted to Fe^2^⁺ by six-transmembrane epithelial antigen of prostate 3 (STEAP-3), which possesses iron reductase activity, and is subsequently released into the cytosolic labile iron pool (LIP) through divalent metal transporter 1 (DMT-1). Fe^2^⁺ is either stored in the labile iron pool or sequestered by ferritin, which is composed of ferritin light chain (FTL) and ferritin heavy chain-1 (FTH-1).FTH-1 exhibits ferroxidase activity, facilitating the conversion of Fe^2^⁺ to Fe³⁺ and subsequently storing it within ferritin molecules, which helps decrease the concentration of free iron. Iron export from cells to the extracellular space is facilitated by the membrane protein solute carrier family 40 member 1 (SLC40A1, or ferroportin 1, FPN1) and lipocalin-2 (LCN-2), which oxidizes Fe^2^⁺ to Fe³⁺. Moreover, studies have enhanced our understanding of how hepcidin regulates systemic iron balance. Hepcidin is an essential peptide hormone responsible for controlling iron levels in the body by regulating its absorption and release (Sangkhae and Nemeth, 2017; Qian et al., 2013). Furthermore, ferritinophagy has been recognized as a vital regulator of intracellular iron homeostasis and is significantly involved in ferroptosis. This process involves the degradation of ferritin via the autophagy pathway to release stored iron, thereby influencing iron availability and the occurrence of ferroptosis (Rochette et al., 2022; Arosio, 2022). In ferroptosis research, a new role for the ATM protein has been identified. Screening of protein kinases associated with ferroptosis highlights the critical function of ATM in maintaining iron balance (Chen et al., 2020). Cancer cells demonstrate elevated levels of intracellular iron, which not only enhances cell growth and proliferation but also triggers ferroptosis via the generation of ROS (Shen et al., 2021; Yang et al., 2020). The use of iron-based nanoparticles and magnetic fields in cancer therapy promotes ferroptosis by regulating cellular iron concentrations, opening new pathways for cancer treatment (Wang et al., 2018). Under high-iron conditions, iron treatment induces ferroptosis in Slc7a11^−/−^ cells, highlighting the critical role of iron in initiating ferroptosis mediated by SLC7A11. This indicates its promise as a viable treatment target for addressing tissue damage linked to hemochromatosis (Wang et al., 2017).

The maintenance of iron balance is also mediated by various iron-modulating proteins. Iron-responsive element-binding protein-2 (IREB-2) reduces intracellular iron levels and prevents ferroptosis by upregulating FTL and FTH-1 (Yao et al., 2021). Heme oxygenase-1 (HO-1) contributes to ferroptosis through the elevation of intracellular iron and the promotion of lipid peroxidation (Kwon et al., 2015). Nuclear receptor coactivator 4 (NCOA4) promotes the liberation of intracellular iron by enhancing ferritin degradation (Santana-Codina and Mancias, 2018). Nuclear factor erythroid 2-related factor 2 (Nrf2) impacts ferroptosis through the modulation of genes with antioxidant response elements (ARE) (Yan R. et al., 2023). When intracellular iron balance is compromised, surplus iron produces ROS via the Fenton reaction, resulting in lipid peroxidation and initiating ferroptosis. Thus, maintaining iron homeostasis is essential for ferroptosis.

### 2.3 Lipid peroxidation

During ferroptosis, lipid peroxidation plays a pivotal role, marked by the buildup of lipid peroxides that cause cytotoxic effects and trigger the cell death pathway. Lipid peroxidation involves the removal of electrons from membrane lipids by reactive species, such as reactive oxygen species, peroxyl radicals, and hydroxyl radicals. This ultimately causes the oxidation of polyunsaturated fatty acids (PUFAs) in cell membranes.Specifically, PUFAs are acylated by acyl-CoA synthetase long-chain family member 4 (ACSL-4) to generate PUFA-CoA. This compound subsequently reacts with phosphatidylethanolamine (PE) through the action of lysophosphatidylcholine acyltransferase 3 (LPCAT-3) to form PUFA-PE.Lipoxygenase (LOX) is essential for oxidizing PUFA-PE, facilitating the production of lipid hydroperoxides (PUFAs-OOH), which are vital for ROS generation. Diminishing the activity of ACSL-4 and LOX can significantly hinder the synthesis and function of PUFA-PE, thereby attenuating ferroptosis. In breast cancer cell models, the disruption of the ACSL-4 gene significantly reduces PUFA-PE production and inhibits ferroptosis (Xu et al., 2024). Additionally, thiazolidinedione drugs and 5-LOX inhibitors such as Zileuton can also inhibit ferroptosis by specifically targeting ACSL-4 and LOX (Jiao et al., 2020; Chen et al., 2018).

12/15-LOX is essential in the lipid oxidation pathway associated with ferroptosis,and its inhibition can significantly suppress ferroptosis (Yang et al., 2016). Furthermore, peroxisomal enzymes are also crucial in the ferroptosis process.Alkylglycerone phosphate synthase, fatty acyl-CoA reductase 1 (FAR1), and glyceronephosphate O-acyltransferase (GNPAT) are essential for the pathway that is independent of ACSL4 and LPCAT3. These enzymes facilitate the buildup of lipid peroxides via various processes (Mao et al., 2021; Lee et al., 2021).

Recent research has identified the involvement of various lipid peroxidation pathways in ferroptosis. These pathways are essential for the execution of ferroptosis, operating through diverse oxidative mechanisms, product profiles, and lipid electrophiles (Do and Xu, 2023; Endale et al., 2023; Van Kessel et al., 2022). ROS induce ferroptosis by oxidizing the phospholipid bilayer, leading to lipid peroxidation (Endale et al., 2023). Mitochondrial lipid peroxidation is a key contributor to ferroptosis.Employing mitochondria-targeted antioxidants and reductants aimed at electron transport chain complex I can prevent cell death (Lyamzaev et al., 2023).

In conclusion, lipid peroxidation is essential to the mechanism of ferroptosis.Regulating the activity of ACSL-4, LPCAT-3, LOX, and related pathways is an important strategy for inhibiting ferroptosis.

### 2.4 Voltage-sensitive anion channel (VDAC) signaling pathway

VDAC functions as a channel protein located in the outer mitochondrial membrane, facilitating the transport of ions and metabolites. It comprises three isoforms—VDAC-1, VDAC-2, and VDAC-3—which play a role in mediating metabolite exchange between mitochondria and different cellular compartments. In addition to its function in managing mitochondrial metabolism and energy production, VDAC may affect cell viability and apoptotic processes through interactions with a variety of ligands and proteins (Heslop et al., 2021; Shoshan-Barmatz et al., 2010; Shoshan-Barmatz and Golan, 2012).

Erastin, a well-known ferroptosis inducer, can stimulate VDAC when microtubules are present, leading to increased mitochondrial membrane potential. This leads to the generation of ROS, impaired mitochondrial function, and subsequent cell death (Yang et al., 2016). The activation of the mitochondrial permeability transition pore (mPTP) can increase the production of ROS in mitochondria, disrupt membrane potential, and cause ATP depletion, ultimately triggering cell death via either programmed or non-programmed pathways (Alano et al., 2004). There is a close connection between VDAC and mPTP. The opening of VDAC may lead to elevated mitochondrial calcium ion levels, subsequently triggering mPTP activation. This process results in a decline in mitochondrial transmembrane potential and the generation of substantial levels of ROS, which further leads to mitochondrial dysfunction and cell death (Xie et al., 2016).

Studies have demonstrated that Erastin induces ferroptosis by activating VDAC, thereby affecting the permeability of the mitochondrial outer membrane (Yang et al., 2016). VDAC and mPTP work synergistically in the mechanism of ferroptosis. The activation of VDAC and the opening of mPTP jointly promote the process of ferroptosis (Xie et al., 2016). Research has indicated that the VDAC pathway is essential for regulating cancer metabolism and may be targeted to enhance cancer cell death, contributing to the development of new anticancer therapies (Heslop et al., 2021).

Consequently, the VDAC pathway is pivotal in the ferroptosis process. A comprehensive grasp of the mechanisms underlying the VDAC pathway not only sheds light on the biological foundations of ferroptosis but also presents potential targets for the development of innovative cancer treatments focused on inducing ferroptosis.

### 2.5 Dysregulation of the antioxidant system

#### 2.5.1 GSH-GPX4 signaling pathway

The GSH-GPX4 signaling pathway is essential for maintaining intracellular redox balance and regulating ferroptosis. System Xc-operates as a membrane-bound amino acid transporter in mammalian cells, consisting of a heterodimer formed by SLC7A11 and SLC3A2, where SLC7A11 is the primary component.System Xc-facilitates the exchange of extracellular cystine (Cys) for intracellular glutamate (Glu) at a 1:1 ratio, supplying the necessary precursors for the synthesis of intracellular glutathione (GSH). The transport of cystine is a crucial step in the production of GSH.

GPX4 is a GSH-dependent antioxidant enzyme that transforms GSH into oxidized glutathione (GSSG), effectively neutralizing ROS produced during cellular respiration and metabolic processes, thus playing a crucial role in preventing lipid peroxidation. Both GSH and selenium (Se) are essential for preserving the functionality and activity of GPX4. When GPX4 activity is impaired, it typically leads to reduced levels of GSSG and a significant increase in ROS, ultimately triggering ferroptosis.

In the context of HT-1080 cells, silencing the SLC7A11 gene with siRNA significantly increased the cells’ vulnerability to ferroptosis triggered by erastin, whereas enhancing the expression of SLC7A11 improved their resistance to this type of cell death (Chang et al., 2018). Erastin, a representative inducer of ferroptosis, directly inhibits System Xc-, depleting intracellular cystine and reducing GSH biosynthesis. This results in a decrease in GPX4 activity, an increase in lipid peroxide accumulation, and ultimately leads to ferroptosis. RSL3, another trigger of ferroptosis, covalently alters selenocysteine, directly inhibiting GPX4 and resulting in a buildup of intracellular lipid peroxides, which ultimately causes cell death (Li F.-J. et al., 2022).

Research indicates that various small molecules, including FINO2, FIN56, ML162, ML210, and buthionine sulfoximine, inhibit GPX4 via distinct mechanisms, thereby promoting ferroptosis (Tang et al., 2021; Gaschler et al., 2018; Hu et al., 2021). For example, FINO2 triggers ferroptosis by directly oxidizing iron and suppressing GPX4 activity (Li F.-J. et al., 2022). Selenium plays a dual role by contributing to the synthesis of GPX4 through the formation of selenocysteine (Sec), the 21st amino acid, and by promoting GPX4 expression via the activation of transcription factors TFAP2c and Sp1 (Xing et al., 2022). The mevalonate (MVA) pathway contributes to GPX4 synthesis by facilitating the production of selenocysteine tRNA, primarily generating isopentenyl pyrophosphate (IPP) and coenzyme Q10 (CoQ10) as key products (Kato et al., 2022).

In conclusion, the GSH-GPX4 signaling pathway is essential for regulating ferroptosis, and altering this pathway can greatly impact cell survival and apoptosis.The core mechanisms of this pathway involve cystine uptake via System Xc-, GSH synthesis, and GPX4 activity maintenance. Disruption of these processes results in increased intracellular ROS and lipid peroxidation, ultimately inducing ferroptosis.

#### 2.5.2 FSP1-CoQ10 signaling pathway

The FSP1-CoQ10 pathway plays a vital role in the modulation of ferroptosis. Ferroptosis suppressor protein 1 (FSP1), referred to as AIFM2, functions as a cytosolic oxidoreductase. FSP1 utilizes the reducing power of NADPH to convert coenzyme Q10 (CoQ10) into its active form. NADPH acts as a vital reducing agent, supplying the necessary electrons for FSP1 to transform CoQ10 into a highly effective lipophilic antioxidant that captures free radicals. The active form of CoQ10 effectively intercepts and neutralizes lipid peroxidation products, safeguarding cell membranes from damage and preventing cell death, thus playing a key role in inhibiting ferroptosis. This mechanism is vital for sustaining the redox state within cells and defending them against oxidative stress.

Studies indicate that the FSP1-CoQ10 pathway can mitigate the impacts of ferroptosis inducers like erastin and RSL3 by decreasing ROS and lipid peroxide levels, thereby safeguarding cells from death.For instance, a recent investigation revealed that cells deficient in FSP1 exhibited markedly lower CoQ10 levels, coupled with significantly elevated lipid peroxides and ROS, underscoring the essential function of FSP1 in modulating CoQ10 and inhibiting ferroptosis (Lv et al., 2023b). Another investigation demonstrated that the overexpression of FSP1 notably elevates intracellular CoQ10 levels, decreases lipid peroxide formation, and thus enhances cell survival against ferroptosis (Gong et al., 2023). Additionally, FSP1 influences disease prognosis and treatment by regulating the redox cycling of CoQ10 and vitamin K (Li W. et al., 2023).

Recent research has underscored the critical role of the FSP1-CoQ10 pathway in the regulation of ferroptosis, indicating possible directions for future therapeutic strategies. Evidence indicates that inhibiting FSP1 is crucial for promoting ferroptosis in cancer cells and overcoming treatment resistance. Compounds such as FSEN1 have been identified as selective inhibitors of FSP1, increasing the vulnerability of cancer cells to ferroptosis (Hendricks et al., 2023).

In conclusion, there is an increasing body of research focused on the significance of the FSP1-CoQ10 pathway in ferroptosis. For example, experiments using small molecule compounds to regulate this axis to inhibit ferroptosis have yielded positive results (Wei et al., 2020). These specific research findings not only underscore the significance of the FSP1-CoQ10 axis in cellular protection but also offer fresh perspectives for designing novel antioxidants.

#### 2.5.3 GCH1-BH4 signaling pathway

The GCH1-BH4 signaling pathway is essential for the regulation of ferroptosis.GTP cyclohydrolase 1 (GCH1) serves as the rate-limiting enzyme in the production of tetrahydrobiopterin (BH4), facilitating the transformation of GTP into BH4.As a key antioxidant, BH4 specifically prevents the autoxidation of phospholipids rich in polyunsaturated fatty acids, thereby preventing lipid peroxidation. Additionally, BH4 enhances the synthesis of coenzyme Q10 (CoQ10), scavenges free radicals, and safeguards cellular membranes against oxidative injury.

Lipid peroxidation is integral to the ferroptosis process. BH4 inhibits ferroptosis by halting the production of lipid peroxides.For instance, research indicated that in a GCH1 knockdown mouse model, decreased levels of BH4 resulted in a marked rise in lipid peroxidation and the induction of ferroptosis (Kraft et al., 2019). Additionally, Investigations have indicated that a mouse model with GCH1 overexpression leads to significantly elevated BH4 levels, reduced lipid peroxide formation, and enhanced cellular resistance to ferroptosis (Hu et al., 2022).

Specifically, in one model, researchers discovered that downregulating GCH1 expression through gene knockout technology resulted in a notable reduction in BH4 levels in mice, accompanied by a marked increase in lipid peroxides and ferroptosis markers (Hu et al., 2022). On the other hand, upregulating GCH1 using gene overexpression technology significantly increased BH4 levels in cells and reduced oxidative stress and ferroptosis. This effect was particularly notable in certain neurodegenerative disease models (Wei et al., 2020).

These findings not only highlight the essential function of the GCH1-BH4 signaling cascade in regulating ferroptosis but also offer new potential targets for future therapeutic interventions. Understanding the mechanisms of this pathway can offer an important theoretical foundation for developing new anti-ferroptosis treatment strategies. For example, a recent study found that using a drug called tetrahydrobiopterin supplement significantly reduced ferroptosis and alleviated myocardial damage in a MIRI model by modulating the GCH1-BH4 pathway (Rochette et al., 2022). This discovery opens up new perspectives and potential avenues for clinical interventions.

#### 2.5.4 DHODH-CoQ signaling pathway

The DHODH-CoQ signaling pathway is essential in modulating ferroptosis.Dihydroorotate dehydrogenase (DHODH), located in the inner mitochondrial membrane, is crucial for synthesizing pyrimidine nucleotides. It facilitates the transformation of dihydroorotate (DHO) into orotate (OA), which is a vital step in the synthesis of pyrimidine nucleotides. During this process, DHODH transfers electrons to coenzyme Q (CoQ), reducing it to ubiquinol (CoQH2), which has antioxidant properties. As a strong free radical-scavenging antioxidant, CoQH2 effectively captures and neutralizes lipid peroxides, protecting cell membranes from damage and cell death, thus playing a critical role in the inhibition of ferroptosis.

Studies indicate that the inhibition of DHODH results in a reduction of CoQH2 levels, which elevates ROS production and subsequently triggers ferroptosis. For instance, a study showed that inhibiting DHODH in cell lines substantially lowered CoQH2 levels, leading to a significant rise in lipid peroxides and ROS, which consequently heightened the incidence of ferroptosis (Xu W. et al., 2021).Conversely, upregulating DHODH or supplementing CoQ can increase CoQH2 levels, reduce ROS production, and thereby enhance cellular resistance to ferroptosis. For example, another study demonstrated that supplementing with CoQ10 could restore the decreased antioxidant capacity caused by DHODH inhibition and reduce ferroptosis (Lyu et al., 2022).

Additionally, recent studies have discovered several new small molecules that can modulate the DHODH-CoQ signaling pathway, thereby influencing ferroptosisFor instance, the use of DHODH inhibitors alongside cisplatin promotes ferroptosis in cervical cancer cells, showcasing synergistic therapeutic effects through the modulation of the mTOR signaling pathway (Jiang et al., 2023). Furthermore, research has revealed that the DHODH mutant A58T shows resistance to the inhibitors Brequinar and BAY2402234, yet retains the ability to bind both drugs and CoQ concurrently (Pokhriyal et al., 2024).

These findings highlight the essential function of the DHODH-CoQ signaling pathway in regulating ferroptosis and suggest potential targets for upcoming therapeutic approaches. Understanding the mechanisms of this pathway can offer an important theoretical foundation for developing new anti-ferroptosis treatment strategies. Recent research indicates that employing gene editing techniques to modulate DHODH expression allows for precise control over ferroptosis, opening new avenues for targeted therapies (Wang et al., 2023).

### 2.6 Ferroptosis and different forms of cellular death

To highlight the differences between ferroptosis and other cell death modalities, the table below (Table 1) summarizes the key characteristics, induction mechanisms, and biomarkers distinguishing ferroptosis from apoptosis, autophagic cell death, necrosis, cuproptosis, and pyroptosis.

**TABLE 1:** Ferroptosis and different forms of cellular death.

	Ferroptosis (Dixon et al., 2012a; Li et al., 2022b)	Apoptosis (Kopacz et al., 2021)	Autophagy (Das et al., 2012)	Necrosis (Li et al., 2021a)	Cuproptosis (Tsvetkov et al., 2022)	Pyroptosis (Zhang et al., 2024)
Cellular Morphological Characteristics	Typical nuclei; Mitochondria exhibiting shrinkage and condensed membranes; Decreased or absent mitochondrial crista densities; Disruption of the outer mitochondrial membrane	Cell shrinkage; Chromatin condensation; Formation of apoptotic bodies; Plasma membrane blebbing; Nuclear fragmentation	Double-membrane vesicles; Engulfed cellular components	Cell swelling; Plasma membrane rupture; Cytoplasmic leakage; Organelle breakdown	Protein aggregation; Loss of iron-sulfur clusters	Cell swelling; Plasma membrane rupture; Pore formation via gasdermin proteins; Release of inflammatory cytokines; Formation of pyroptotic bodies
Induction Mechanisms	Regulation of Iron Metabolism; Lipid Peroxidation; Dysregulation of the Antioxidant System	Caspase activation; Mitochondrial outer membrane permeabilization (MOMP); Death receptor signaling	Nutrient deprivation; mTOR inhibition	ROS production; Mitochondrial dysfunction; Calcium overload; Inflammation; ATP depletion	Copper binding to lipoylated TCA cycle proteins	Inflammasome Activation; Caspase-1 Activation; Caspase-4/5/11 Activation; Gasdermin D Cleavage; Lipopolysaccharide (LPS) Response
Key Biomarkers	GPX4,ACSL4	Caspase-3, -7, -8, -9; Bcl-2 family proteins (Bax, Bak, Bcl-2, Bcl-XL); Cytochrome c release	LC3-II/I ratio, p62 degradation	HMGB1 release; LDH release; Propidium iodide uptake; Phosphorylation of RIPK1 and RIPK3	FDX1; Lipoylated proteins (LIAS, DLAT)	IL-1β, IL-18, GSDMD cleavage
Reversibility	Irreversible	Reversible (early stage)	Reversible (early stage)	Irreversible	Irreversible	Irreversible
Typical Inhibitors	Ferrostatin-1; Deferoxamine; Liproxstatin-1	Z- VAD-FMK AA- BB- Q-VD-OPh CC- Rapamycin	Chloroquine; Bafilomycin A1	Necrostatin-1 GSK′872 Necrosulfonamide	Tetrathiomolybdate	Necrosulfonamide U0126 Bay-117082 MCC950; VX-765

In this section, we will provide a table highlighting the distinctions between ferroptosis and other forms of cell death, which will facilitate a better understanding of the unique features of ferroptosis.

## 3 The impact of ferroptosis on MIRI

### 3.1 Overview

Coronary artery blockage can cause myocardial ischemia, presenting as intense and prolonged chest pain, and potentially leading to myocardial infarction, shock, arrhythmias, or heart failure. Typical treatment options encompass coronary angioplasty, percutaneous coronary intervention (PCI), and coronary artery bypass grafting (CABG). These procedures aim to quickly restore circulation to the affected region, thereby ensuring the delivery of oxygen and nutrients to the myocardium, protecting compromised heart tissue, and improving patient survival rates. MIRI describes a condition where cardiac function worsens instead of improving during reperfusion after myocardial ischemia. Despite the critical role of reperfusion in restoring blood and oxygen supply, it also triggers a series of complex pathophysiological responses.

**FIGURE 2:**
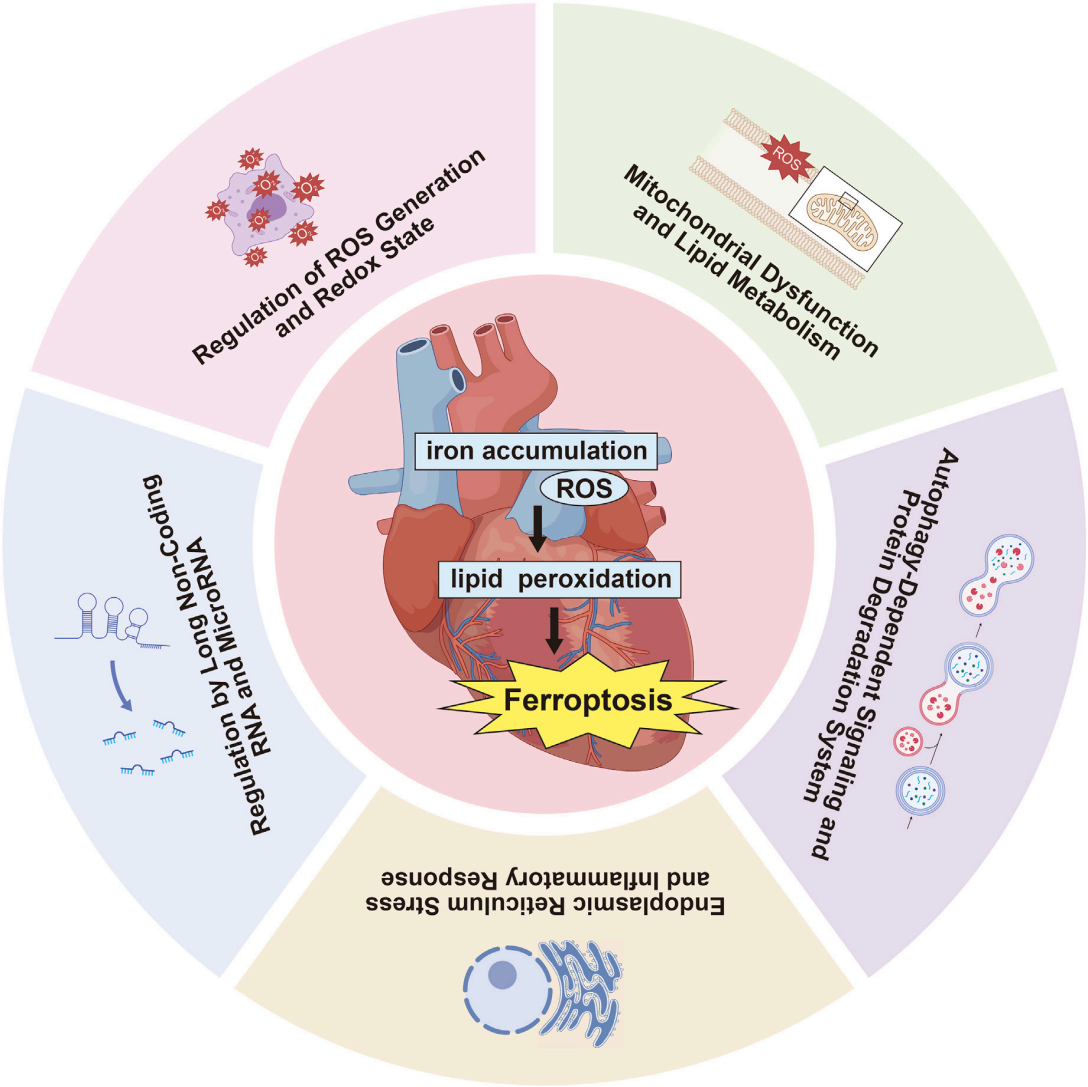
Recent research on ferroptosis in MIRI highlights several critical aspects.

Numerous studies have investigated the impact of ferroptosis on MIRI, concentrating on several key areas: ROS production and redox state regulation, mitochondrial dysfunction and lipid metabolism, autophagy-related signaling and protein degradation mechanisms, endoplasmic reticulum stress (ERS) and inflammatory reactions, along with the influence of long non-coding RNAs (lncRNAs) and microRNAs in ferroptosis pathways (Table 2, Figure 2). Oxidative stress disrupts cellular homeostasis, interferes with cell division and differentiation, and impairs cellular signaling, ultimately leading to cell membrane rupture, cellular swelling, or cell death. Oxidative stress plays a crucial role in reperfusion injury, not only leading to cellular harm but also being intimately linked with ferroptosis, a type of iron-dependent programmed cell death.

**TABLE 2:** Key aspects of ferroptosis in MIRI: Recent research insights.

Key aspect	References	Findings	Pathway or key factor
ROS production and redox state regulation	Cai et al. (2023)	15-HpETE degrades Pgc1α, causing mitochondrial dysfunction, lipid peroxidation, and worsened ferroptosis in MIRI.	Alox15/15-HpETE; Pgc1α
	Li et al. (2020)	Ferroptosis drives MIRI in diabetes, with key roles for ROS and GSH depletion	GSH; ERS
	Li et al. (2021b)	Inhibition of DNMT-1 reduces ferroptosis in diabetic MIRI by decreasing NCOA4-mediated ferritinophagy	DNMT-1; NCOA4; GPX4
	Ishimaru et al. (2024)	Deferasirox combined with CsA reduces MIRI by targeting ferroptosis, leading to smaller infarcts and better cardiac remodeling	Deferasirox; Cyclosporine A
	Qian et al. (2023)	CsA-loaded apoferritin alleviates MIRI by inhibiting ferroptosis, reducing iron/lipid peroxidation, and restoring mitochondrial function	ACSL4; GPX4
	Yang et al. (2023b)	Ferroptosis occurs during reperfusion, not ischemia; Fer-1 alleviates MIRI, with Sesn1 playing a protective role	GPX4; FTH1; Sestrin 1
	Paraskevaidis et al. (2005)	Deferoxamine during CABG reduces lipid peroxidation and improves myocardial recovery, especially in patients with low ejection fractions	ROS; Free Radicals
	Williams et al. (1991)	Deferoxamine improves myocardial recovery by reducing reperfusion-induced oxygen radicals during ischemia	ROS; Deferoxamine
	Tian et al. (2022)	NRF2/FPN1 activation alleviates MIRI in diabetic rats by regulating iron and inhibiting ferroptosis	NRF2; FPN1
	Zhao et al. (2024)	Zhilong Huoxue Capsule alleviates MIRI by inhibiting ferroptosis via PI3K/AKT/Nrf2 and suppressing ACSL4	PI3K/AKT; Nrf2; HO-1; GPX4; ACSL4
Mitochondrial dysfunction and lipid metabolism	Yamada et al. (2024)	Mitochondrial dysfunction from 15-LOX/PEBP1-triggered ferroptosis activates PINK1/Parkin mitophagy to reduce oxidative stress	15-LOX/PEBP1; PINK1/Parkin; GPX4
	Yang et al. (2023a), Zhou et al. (2024), Zhong et al. (2024), Guo et al. (2024), Xu et al. (2021b), Yan et al. (2023b)	Some drugs reduces MIRI by inhibiting ferroptosis via the Nrf2/GPX4 pathway	Nrf2/GPX4; Iron overload
	Hu et al. (2024a)	Activation of PPAR-α mitigates MIRI by inhibiting ferroptosis and mitochondrial damage through 14-3-3η upregulation	PPAR-α/14-3-3η Pathway
	Zhang et al. (2021)	Polydopamine nanoparticles inhibit ferroptosis and protect against MIRI by scavenging ROS and chelating Fe^2^⁺	ROS; Fe^2^⁺ Chelation
	Liao et al. (2023)	SIRT1-SIRT3 axis abnormalities promote MIRI through ferroptosis by silencing the PINK1/Parkin pathway	SIRT1-SIRT3; PINK1/Parkin
	Li et al. (2022c)	Propofol protects against MIRI by inhibiting ferroptosis via the AKT/p53 signaling pathway	AKT/p53; GPX4
	Fan et al. (2021)	Baicalin protects against MIRI by inhibiting ferroptosis through ACSL4 suppression and reducing lipid peroxidation	ACSL4; GPX4; Iron Accumulation
Autophagy-related signaling and protein degradation mechanisms	Chen et al. (2021a)	FOXC1 activates ELAVL1 to promote ferroptosis and myocardial injury by regulating autophagy	FOXC1/ELAVL1; Autophagy
	Junhong et al. (2024)	Epicatechin protects against MIRI by reducing ferroptosis via USP14-mediated autophagy inhibition	USP14 Pathway; NCOA4; ROS
	Qiu et al. (2023)	YAP reduces ferroptosis in MIRI by promoting NEDD4L-mediated degradation of ACSL4	YAP/NEDD4L/ACSL4 Axis
	Ma et al. (2020)	USP22 protects against MIRI by inhibiting ferroptosis through the SIRT1/p53/SLC7A11 axis	SIRT1/p53/SLC7A11
	Hu et al. (2024b)	EGCG protects against MIRI by inhibiting ferroptosis, apoptosis, and autophagy through 14-3-3η modulation	14-3-3η Pathway; GPX4; PTGS2
ERS and inflammatory reactions	Li et al. (2020)	Ferroptosis involvement in diabetes MIRI is mediated through ERS.	ATF4/CHOP; GPX4
	Jiang et al. (2024)	CBX7 downregulation reduces ferroptosis and ERS, protecting against MIRI.	CHOP/GRP78; SLC7A11/GPX4
	Huang et al. (2023)	Ferroptosis and ERS contribute to intermittent hypoxia-induced myocardial injury, alleviated by N-acetylcysteine	GPX4/xCT; NOX4/PERK/CHOP
	Su et al. (2024)	Inhibition of circ_0073932 attenuates MIRI via miR-493-3p/FAF1/JNK signaling	miR-493-3p/FAF1/JNK
Influence of lncRNAs and microRNAs	Sun et al. (2022b)	LncAABR07025387.1 aggravates MIRI by promoting ferroptosis through the miR-205/ACSL4 axis	miR-205/ACSL4
	Zhang et al. (2022a)	Exosomal lncRNA Mir9-3 hg mitigates ferroptosis in cardiomyocytes during ischemia-reperfusion injury through the Pum2/PRDX6 pathway	Pum2/PRDX6; GPX4
	Dai et al. (2023)	AC005332.7 attenuates ferroptosis in myocardial infarction through modulation of the miR-331-3p/CCND2 pathway	miR-331-3p/CCND2; GPX4/ACSL4
	Gao et al. (2022)	Downregulation of lncRNA Gm47283 alleviates myocardial infarction through the miR-706/Ptgs2/ferroptosis pathway	miR-706/Ptgs2
	Yu et al. (2024)	Silencing of NEAT1 reduces ferroptosis in myocardial infarction by modulating the miR-450b-5p/ACSL4 axis	miR-450b-5p/ACSL4
	Sun et al. (2021a)	miR-135b-3p exacerbates myocardial I/R injury by promoting ferroptosis via GPX4 inhibition	miR-135b-3p; GPX4
	Ji et al. (2023)	miR-196c-3p with NIR-II light-triggered gel inhibits ferroptosis in MIRI by targeting NOX4, P53, LOX.	miR-196c-3p; NOX4/P53/LOX; GPX4
	Yu et al. (2023)	EGCG mitigates acute myocardial infarction through the suppression of ferroptosis via the miR-450b-5p/ACSL4 pathway	miR-450b-5p/ACSL4; SLC7A11; GPX4
	Lei et al. (2022)	Exosomal miR-210-3p protects cardiomyocytes by inhibiting ferroptosis via the TFR pathway	miR-210-3p/TFR; GPX4
	Ye et al. (2023)	Ginsenoside Re protects against MIRI-induced ferroptosis via miR-144-3p targeting SLC7A11	miR-144-3p/SLC7A11; GSH
	Zhu et al. (2024)	DEX mitigates ferroptosis in cardiomyocytes via miR-141-3p/lncRNA TUG1 regulation	miR-141-3p/lncRNA TUG1; GPX4; ACSL4

Research evidence specifically indicates that ferroptosis is a key factor in MIRI.For instance, research indicates that ferroptosis occurs during the myocardial reperfusion phase, leading to cardiac injury through mechanisms associated with ROS and iron accumulation (Yang F. et al., 2023). Another study indicated that circHMGA2 aggravates MIRI by enhancing ferroptosis and pyroptosis through the suppression of the NLRP3 pathway (Feng et al., 2023). Furthermore, research has suggested a potential link between acetylation modifications and ferroptosis in the context of MIRI (Yang Y. et al., 2023).

These discoveries lay the groundwork for deeper investigation into the mechanisms driving ferroptosis in MIRI.

### 3.2 Key Aspects of Ferroptosis in MIRI

#### 3.2.1 Regulation of ROS generation and redox state

Oxidative stress is considered a central pathogenic mechanism in MIRI, linked to the increased generation of ROS during the reperfusion period. The over accumulation of ROS results in lipid peroxidation of membranes, compromising the barrier function of cell membranes. The antioxidant system preserves redox balance by regulating intracellular ROS levels and managing the interplay between normal cellular metabolism and disease-related pathways.Upregulating antioxidant enzyme expression can safeguard tissues against damage induced by oxidative stress following myocardial reperfusion, thereby offering cardioprotective effects. A key pathogenic mechanism contributing to MIRI is the swift increase in ROS that takes place upon restoration of blood flow and reoxygenation of myocardial tissues (Cai et al., 2023).

ROS are pivotal in the ferroptosis associated with MIRI. Ferroptosis is initiated by elevated intracellular iron concentrations and diminished levels of the antioxidant glutathione (GSH), resulting in increased ROS levels. This increase in ROS causes lipid peroxidation, culminating in cell death (Li et al., 2020). ROS are generated via non-enzymatic pathways when iron ions are present. For example, the Fenton reaction in the labile iron pool produces Fe³⁺ and hydroxyl radicals from free iron ions, Fe^2^⁺, and H₂O₂ (Li W. et al., 2021). Elevated ROS levels can be mitigated by the iron chelator deferoxamine, which decreases lipid peroxidation and reduces the incidence of ferroptosis (Ishimaru et al., 2024). Conversely, increased expression of iron transport proteins can enhance iron-induced ROS production, ultimately triggering ferroptosis (Qian et al., 2023). Research indicates that liproxstatin-1 markedly suppresses ferroptosis and mitigates MIRI by limiting lipid peroxidation-induced ROS buildup, maintaining mitochondrial integrity, elevating GPX4 protein levels, and decreasing overall ROS levels (Yang F. et al., 2023).

Research indicates that in individuals with coronary artery disease (CAD) undergoing CABG surgery, intravenous administration of deferoxamine 8 hours post-operation can markedly reduce the generation of ROS and safeguard the heart from injury caused by reperfusion. This approach is especially advantageous for patients with diminished left ventricular ejection fraction (LVEF) (Paraskevaidis et al., 2005).Furthermore, during the initial perfusion phase of isolated rabbit hearts, administering a particular dose of deferoxamine, an iron chelator, significantly lowers free radical production caused by reperfusion, leading to cardioprotective outcomes (Williams et al., 1991).

Regulation of redox states is also crucial in MIRI. Nrf2 is crucial in mitigating oxidative stress by controlling the expression of antioxidant enzymes and preserving intracellular redox homeostasis. Activation of Nrf2 can alleviate myocardial damage caused by MIRI (Tian et al., 2022). Additionally, studies have indicated that Zhilong Huoxue Tongyu capsule reduces MIRI-induced ferroptosis by upregulating Nrf2 via the PI3K/AKT signaling pathway and triggering the activation of the HO-1/GPX4 pathway (Zhao et al., 2024).

These comprehensive studies have given us deeper insights into the crucial roles that ROS production and redox state regulation play in ferroptosis and MIRI.This information offers important scientific support for the development of future treatment approaches.

#### 3.2.2 Mitochondrial dysfunction and lipid metabolism

The mitochondria serve as the main source of ROS within cells. During MIRI, mitochondrial dysfunction leads to decreased efficiency in the electron transport system and increased electron leakage, culminating in an overproduction of RO. The overproduction of ROS triggers intracellular oxidative stress, which results in cellular damage and death (Chen et al., 2022). Mitochondrial dynamics, including processes like fusion and fission, are essential for modulating mitochondrial activity and sustaining cellular energy homeostasis. Abnormal alterations in mitochondrial morphology frequently accompany mitochondrial dysfunction, impacting metabolic performance and ROS generation, which can lead to ferroptosis (Li J. et al., 2023).

Mitophagy serves as an essential process for clearing defective mitochondria.Mitochondrial dysfunction frequently occurs alongside impaired mitophagy, resulting in the buildup of damaged mitochondria, elevated ROS levels, and the triggering of ferroptosis (Yamada et al., 2024). Moreover, mitochondria serve as the main hub for intracellular iron metabolism, playing a crucial role in maintaining cellular iron balance. Impaired mitochondrial iron regulation can result in iron overload and increased ROS production, ultimately initiating ferroptosis (Yang T. et al., 2023). Mitochondrial metabolic function directly influences the intracellular redox state. Disruption of mitochondrial metabolism can lead to redox imbalance, thereby increasing ROS production and inducing ferroptosis (Hu et al., 2024a). Finally, mitochondrial dysfunction not only affects mitochondrial metabolism itself but also regulates cell survival and death through interactions with other intracellular signaling pathways.For example, research has demonstrated a close relationship between mitochondrial dysfunction and ferroptosis in MIRI. Mitochondrial impairment can affect ferroptosis via multiple signaling mechanisms (Zhang et al., 2021).

Recent research has identified that compounds like N-acetylcysteine can safeguard the heart against ischemia-reperfusion injury by inhibiting ferroptosis. These small molecules can prevent ferroptosis by targeting mitochondrial dysfunction and decreasing ROS generation (Zhou et al., 2024). Additionally, Ginsenoside Rg3 significantly alleviates ferroptosis in MIRI by activating the keap1/Nrf2/GPX4 signal transduction pathway (Zhong et al., 2024).

PUFAs are crucial elements of cellular membranes and are particularly vulnerable to oxidative degradation. A defining characteristic of ferroptosis is the peroxidation of lipids. Mitochondrial dysfunction may result in lipid metabolism disorders, which heighten the oxidation of PUFAs and consequently trigger ferroptosis (Guo et al., 2024). PUFAs in cell membranes are susceptible to oxidation, leading to the formation of lipid peroxides. These peroxides may disrupt the structural integrity of cell membranes, resulting in cellular dysfunction. In the context of mitochondrial dysfunction, lipid metabolism is disrupted, exacerbating the oxidation of PUFAs and thereby inducing ferroptosis (Liao et al., 2023). Research has revealed that the NRF2 signaling pathway is essential for managing the cellular response to antioxidant stress.NRF2 can activate various antioxidant enzymes, thereby mitigating lipid peroxidation caused by ROS (Xu S. et al., 2021). Fucoxanthin, an algal extract, mitigates MIRI by triggering the NRF2 signaling cascade, which inhibits lipid peroxidation and prevents ferroptosis (Yan J. et al., 2023). Mitochondria are vital in maintaining intracellular iron balance. Imbalance in iron regulation intensifies lipid peroxidation and triggers ferroptosis (Li J.-y. et al., 2021).

Lipid metabolism interacts with multiple intracellular signaling pathways, influencing cell survival and death. The p53 signaling pathway influences ferroptosis through the modulation of genes involved in lipid metabolism (Li S. et al., 2022). Furthermore, the Nrf2 signaling pathway reduces lipid peroxidation and ferroptosis by modulating the levels of antioxidant enzymes (Yan J. et al., 2023). Moreover, inhibitors of ACSL4 and LPCAT3 can safeguard cells against ferroptosis by modulating lipid metabolism and decreasing the oxidation of PUFAs (Fan et al., 2021).

These investigations provide deeper insights into the pivotal roles of impaired mitochondrial function and lipid metabolism in ferroptosis and MIRI.

#### 3.2.3 Autophagy-dependent signaling and protein degradation system

Autophagy is a self-degradation mechanism in cells that facilitates the removal of damaged organelles and proteins, thereby recycling valuable components. This process is crucial for maintaining cellular homeostasis and addressing stress and damage within the cell. Autophagy consists of several critical stages: the formation of autophagosomes, their merging with lysosomes, and the later degradation and repurposing of cellular components (Das et al., 2012).

Autophagy is crucial in the process of ferroptosis. It can modulate intracellular iron distribution and oxidative stress levels, thus influencing the onset of ferroptosis. Specifically, autophagy degrades iron-containing proteins (such as ferritin and iron-containing enzymes), releasing free iron. When autophagy is excessively active, it results in elevated intracellular free iron levels. This iron contributes to the production of increased ROS, leading to lipid peroxidation. The ensuing damage to cell membranes ultimately causes cell death through ferroptosis (Chen H.-Y. et al., 2021). Therefore, autophagy significantly influences ferroptosis by modulating iron homeostasis and redox balance.

In MIRI, autophagy-dependent ferroptosis is viewed as a crucial process causing myocardial injury and impaired function.Research indicates that ELAVL1 enhances the autophagic process and affects ferroptosis by stabilizing autophagy-related gene mRNA and boosting their expressionIn the MIRI model, FOXC1 modulates the regulation of genes related to autophagy, thereby impacting the onset and development of the autophagic process. This regulation affects the intracellular distribution of iron and oxidative stress levels, which is crucial for reducing ferroptosis (Chen H.-Y. et al., 2021). Additionally, during the MIRI process, the degradation of ferritin releases iron, which facilitates iron-driven Fenton reactions, leading to oxidative stress and compromised cardiac function. By regulating the autophagic process, the degradation of ferritin can be influenced, thus influencing the initiation of ferroptosis (Yang and Lin, 2023). A different study suggests that Epicatechin mitigates ferroptosis through the enhancement of autophagy and the suppression of oxidative stress, thereby safeguarding the MIRI (Junhong et al., 2024). This underscores the essential role of autophagy in modulating ferroptosis during MIRI.

The regulation of ferroptosis is significantly influenced by the protein degradation system, which primarily encompasses the ubiquitin-proteolytic system (UPS) and the lysosomal pathway. The UPS maintains intracellular protein homeostasis by tagging and degrading damaged proteins. Research indicates that the UPS plays a vital role in modulating ferroptosis.For instance, YAP enhances the ubiquitination and subsequent degradation of ACSL4 facilitated by NEDD4L, thereby mitigating ferroptosis and offering protective effects in MIRI (Qiu et al., 2023). Likewise, USP22 prevents ferroptosis-mediated cell death in cardiomyocytes via the SIRT1-p53/SLC7A11 signaling pathway, offering additional defense against MIRI (Ma et al., 2020).

Additionally, the interaction between autophagy and the protein degradation system is also crucial in controlling ferroptosis. Research has demonstrated that EGCG shields the heart muscle from ischemia-reperfusion damage by increasing the levels of 14-3-3η protein, which subsequently inhibits autophagy and ferroptosis (Hu et al., 2024b).

Despite some progress, studies focusing on autophagy-related signaling and protein degradation pathways in MIRI remain scarce. The existing research does not fully elucidate their involvement in ferroptosis regulation. Further investigations are required to deepen our understanding of how these mechanisms contribute to MIRI pathology and to explore their therapeutic potential.

#### 3.2.4 Endoplasmic reticulum stress (ERS) and inflammatory response

ERS is a cellular response triggered by various stressors, including disruptions in calcium balance, ROS, and protein misfolding, occurring within the endoplasmic reticulum. The endoplasmic reticulum is essential for the synthesis, folding, and modification of proteins. When its function is compromised, it activates the Unfolded Protein Response (UPR) to reestablish normal endoplasmic reticulum activity.Nonetheless, if the stress persists or is excessively severe, and UPR does not successfully reestablish homeostasis, the cell might initiate apoptosis or other forms of programmed cell death (Li et al., 2020).

ERS has a significant connection with ferroptosis. It can modulate ferroptosis by altering iron metabolism, affecting ROS generation, and regulating the autophagic pathway. Specifically, ERS impacts iron storage and release, thus changing intracellular iron levels. It also increases ROS production, which is crucial for ferroptosis (Jiang et al., 2024).

Studies have demonstrated that ERS triggers ferroptosis by enhancing ROS production and modulating iron metabolism. For example, in the MIRI model, the initiation of the ATF4-C/EBP homologous protein (CHOP) pathway can trigger ERS and promote ferroptosis (Huang et al., 2023). Suppressing ferroptosis can mitigate MIRI induced by ERS.For instance, the application of iron chelators or ferroptosis inhibitors can markedly decrease the levels of markers linked to ERS and ferroptosis, thus mitigating myocardial injury (Chen Y. et al., 2021).

Recent studies have found that inflammation significantly contributes to MIRI induced by ferroptosis. The activation of various inflammatory signaling pathways, including NF-κB and JNK, leads to the production and release of inflammatory factors (Zhang and Kaufman, 2008). These inflammatory factors can additionally intensify ERS and ferroptosis, fostering a detrimental cycle.

ERS is capable of stimulating the NF-κB pathway, which leads to the generation and secretion of pro-inflammatory factors. The activation of NF-κB is strongly associated with the initiation of ferroptosis. A study found that the NF-κB pathway exacerbates myocardial injury in ferroptosis-induced inflammatory responses by increasing ROS and promoting ferritin degradation (Zhao et al., 2023).

Moreover, the JNK pathway is also pivotal in the inflammatory response triggered by ERS and ferroptosis (Ye et al., 2019). ERS regulates the inflammatory response through the JNK pathway, increasing cellular damage. JNK signaling is intimately linked to the secretion of inflammatory mediators during ferroptosis. A study demonstrated that activation of the JNK pathway in the MIRI model leads to myocardial cell death by upregulating ferroptosis-associated genes and the secretion of inflammatory mediators (Su et al., 2024).

These studies highlight the significant function of the inflammatory response in MIRI induced by ferroptosis and reveal the mechanisms by which endoplasmic reticulum stress acts as a key regulatory factor in this process.

#### 3.2.5 Regulation by long non-coding RNA (lncRNA) and MicroRNA

lncRNAs and microRNAs are crucial in regulating gene expression, influencing a range of cellular activities, including ferroptosis (Gao et al., 2023; Xiong et al., 2019). Ferroptosis, a form of regulated cell death reliant on iron and characterized by lipid peroxidation, plays a crucial role in MIRI. This section will explore how lncRNAs and microRNAs regulate ferroptosis and impact the pathophysiology of MIRI.

lncRNAs, which are RNA sequences longer than 200 nucleotides, are involved in modulating gene expression post-transcriptionally. These molecules are crucial for numerous biological processes, including cell differentiation, growth, and apoptosis. Recent studies have underscored the important function of lncRNAs in influencing ferroptosis, a type of regulated cell death, especially in the context of MIRI.

Several lncRNAs have been implicated in ferroptosis regulation during MIRI, acting through diverse molecular pathways. For instance, specific lncRNAs modulate key axes, such as miR-205/ACSL4,miR-450-5p/ACSL4 and miR-706/Ptgs2, influencing the balance between ferroptosis and cardiomyocyte survival (Sun W. et al., 2022; Gao et al., 2022; Yu et al., 2024). Furthermore, some lncRNAs are involved in reducing ferroptosis by interacting with axes like Pum2/PRDX6 or inhibiting key ferroptosis-related genes such as CCND2 (Zhang J.-K. et al., 2022; Dai et al., 2023). Silencing or modulating these lncRNAs offers a promising avenue for mitigating cardiac damage caused by ferroptosis. In summary, lncRNAs are crucial in regulating ferroptosis in MIRI, offering novel therapeutic possibilities for preventing and addressing ischemic heart injury.

MicroRNAs are short non-coding RNAs that modulate gene expression after transcription by interacting with specific mRNAs. In the MIRI, several miRNAs have been implicated in the regulation of ferroptosis. These miRNAs modulate key ferroptosis-related genes, influencing the vulnerability of cardiomyocytes to oxidative stress during MIRI.

For instance, miR-135b-3p has been identified as a direct regulator of GPX4, influencing iron buildup and lipid peroxidation, which are essential processes in ferroptosis. Its altered expression during ischemia-reperfusion worsens cardiac damage by influencing GPX4, as demonstrated in various experimental models of MIRI (Sun et al., 2021b).

Similarly, miR-196c-3p regulates ferroptosis by targeting key genes like NOX4, P53, and LOX, which control oxidative stress in cardiomyocytes. Its manipulation has shown significant effects on ferroptosis markers. Administering miR-196c-3p via a NIR-II light-activated gel mitigated ferroptosis in ischemia-reperfusion models by enhancing GPX4 expression and lowering levels of iron and lipid peroxidation (Ji et al., 2023).

MiRNAs such as miR-450b-5p, miR-210-3p, miR-144-3p, and miR-141-3p are crucial regulators of the balance between antioxidant defenses and ferroptosis in MIRI (Yu et al., 2023; Lei et al., 2022; Ye et al., 2023; Zhu et al., 2024). Targeting these miRNAs offers potential therapeutic strategies to reduce ferroptosis-induced damage.

The role of lncRNAs and microRNAs in regulating ferroptosis during MIRI remains an important area of ongoing research. Future studies should focus on identifying additional RNA molecules linked to MIRI through advanced techniques like high-throughput sequencing, which will further illuminate the complex ferroptosis regulatory network. Examining the specific pathways through which lncRNAs and microRNAs regulate ferroptosis, including the competing endogenous RNA (ceRNA) mechanism, is crucial.With a better understanding of their functions, preclinical experiments can then explore their potential as therapeutic targets for MIRI.

## 4 Prevention and treatment strategies

To prevent and treat MIRI, strategies targeting the suppression of ferroptosis have become a research focus. The table below (Table 3) summarizes some traditional Chinese and Western medicines that have been studied in the past 5 years for protecting against MIRI by inhibiting ferroptosis, along with their research findings.

**TABLE 3:** Treatment strategies of ferroptosis in MIRI.

Drug Name	Year	Research Model	Mechanism of Action	References
Pachymic Acid (PA)	2023	mice/HL-1	Activation through the AMPK pathway	Liu et al. (2024)
Dapagliflozin	2023	rat/H9C2	Targets the MAPK pathway	Chen et al. (2023b)
Puerarin	2023	mice/H9C2	Targets GPX4 and FTH1 pathways	Ding et al. (2023)
Ginsenoside Re	2023	rat/H9C2	Activates the miR-144-3p/SLC7A11 pathway	Ye et al. (2023)
Saponins	2021	mice/H9C2	The activation of the Nrf2/HO-1 pathway	Wang et al. (2021a)
Salvianolic Acid B	2023	rat/H9C2	By reducing GPX4 degradation via the ubiquitin-proteasome system and inhibiting the ROS-JNK/MAPK signaling pathways	Xu et al. (2023)
Dexmedetomidine	2023	H9C2	Via the cAMP/PKA/CREB pathway	Ma et al. (2023)
Hydroxysafflor Yellow A	2023	mice/H9C2	The activation of the HIF-1α/SLC7A11/GPX4 signaling pathway	Ge et al. (2023)
HJ11 Decoction	2022	rat/H9C2	ACSL4-mediated pathway engagement	Zhang et al. (2022b)
Resveratrol	2022	rat/H9C2	Through the USP19/Beclin1-driven autophagy pathway	Li et al. (2022d)
Cyanidin‐3‐Glucoside	2021	rat/H9C2	Via the USP19/Beclin1-mediated autophagy pathway	Shan et al. (2021)
Atorvastatin (ATV)	2022	rat/H9C2	Via activation of the SMAD7/hepcidin pathway	Peng et al. (2022)
Gossypol acetic acid	2021	rat/H9C2	Through the elevation of GPX4 protein expression and reduction of lipid peroxidation	Lin et al. (2021)
Xanthohumol	2022	rat/H9C2	Through regulation of the GPX4 and NRF2 pathways, lowering lipid peroxidation and ROS levels, and binding intracellular iron	Lin et al. (2022)
Geniposide	2022	rat/H9C2	Through activation of the Grsf1/GPx4 axis	Shen et al. (2022)
Isoliquiritigenin	2024	mice/NMCM	Through modulation of the Nrf2/HO-1/SLC7A11/GPX4 axis	Yao et al. (2024a)
Penehyclidine hydrochloride	2023	Rat/H9C2	By decreasing the expression of ACSL4, which results in lower lipid ROS levels	Lin et al. (2023)

## 5 Conclusion and future perspectives

Despite the significant advancements in understanding the role of ferroptosis in MIRI, there remain notable limitations that need to be addressed. Much of the existing research relies on *in vitro* experiments and animal models, which, while useful, fail to fully replicate the intricate complexity of human MIRI. The methodologies employed often utilize isolated cell cultures or small animal models, and while these provide valuable insights, they may introduce biases that limit their relevance to human clinical conditions. Differences in iron regulation, lipid peroxidation, and antioxidant mechanisms between humans and experimental models may contribute to inconsistent therapeutic outcomes, underscoring the urgent need for more clinically relevant models.

In addition, there are still considerable gaps in understanding the precise molecular mechanisms of ferroptosis in MIRI. While it is well established that lipid peroxidation and iron metabolism play key roles in triggering ferroptosis (Baba et al., 2018; Miyamoto et al., 2022), the exact downstream effectors of lipid oxidation and their contribution to cell death remain insufficiently characterized. Similarly, the specific levels of ROS and iron necessary to induce ferroptosis in cardiomyocytes are not well defined. Although initial studies indicate that treatments such as iron chelators and lipid peroxidation inhibitors may have cardioprotective effects, their long-term efficacy and safety profiles have not been comprehensively evaluated in clinical trials, leaving significant room for further investigation (Yang T. et al., 2023; Feng et al., 2019).

Moreover, although ferroptosis has been thoroughly investigated in relation to MIRI, its connections with other types of cell death, such as apoptosis and necrosis, are still not well understood, especially within the wider context of cardiovascular diseases. Exploring the interactions between ferroptosis and various cell death mechanisms may offer a more comprehensive and effective strategy for mitigating cardiomyocyte loss during MIRI. Furthermore, increasing evidence indicates that ferroptosis could be implicated in other cardiovascular disorders, including heart failure and atherosclerosis, paving the way for new research opportunities to explore its wider pathological implications (Mancardi et al., 2021; Yagi et al., 2023; Wang Y. et al., 2021; Meng et al., 2021).

Looking toward future research, it is essential to explore novel therapeutic targets related to ferroptosis. Advanced methodologies like CRISPR-Cas9 gene editing offer a chance to explore under-researched genes related to iron homeostasis and lipid peroxidation (Yao F. et al., 2024; Valashedi et al., 2022; Cheng et al., 2023). These techniques could help identify new regulatory elements that could be targeted for therapeutic intervention. At the same time, the development of more sophisticated and clinically relevant animal models, such as humanized models or larger animals, could help bridge the gap between preclinical studies and human clinical applications. Additionally, standardizing ischemia-reperfusion models and incorporating common comorbidities, such as diabetes and hypertension, would allow for results that better reflect the complexities of real-world clinical conditions.

Additionally, effective implementation of ferroptosis-targeted therapies in clinical settings necessitates extensive randomized controlled trials to assess both immediate advantages and enduring effects on heart function and patient longevity. It is crucial to thoroughly investigate the potential toxicities and side effects of ferroptosis inhibitors, particularly in terms of their effects on iron homeostasis and oxidative stress. Ensuring the safety of these therapies is critical before they can be widely adopted in clinical settings. Since MIRI involves multiple overlapping mechanisms of cell death, a combination of ferroptosis inhibitors with therapies that target apoptosis, necrosis, or inflammation may offer a more comprehensive strategy for managing ischemia-reperfusion injury.

In conclusion, by addressing these research gaps and refining current strategies, ferroptosis-targeted therapies have the potential to significantly improve outcomes not only in MIRI but also in other cardiovascular diseases.Subsequent investigations should prioritize the creation of multi-target strategies that integrate ferroptosis inhibitors with alternative therapies, aiming for a comprehensive approach to address the intricate pathophysiology of ischemia-reperfusion injury.

## Figures and Tables

**Table:** 

**ROS**	Reactive oxygen species
**MIRI**	Myocardial ischemia-reperfusion injury
**GPX4**	Glutathione peroxidase-4
**Alox15**	Arachidonate lipoxygenase 15
**RSL3**	Ras selective lethal compound 3
**Fer-1**	Ferrostatin-1
**VDAC**	Voltage-dependent anion channel
**TF**	Transferrin
**TFR1**	Transferrin receptor 1
**STEAP-3**	Six-transmembrane epithelial antigens of the prostate 3
**DMT-1**	Divalent metal transporter 1
**LIP**	Labile iron pool
**FTL**	The light chain of ferritin
**FTH-1**	The heavy chain of ferritin
**FPN1**	Feroportin-1
**LCN-2**	Lipocalin-2
**ATM**	Ataxia Telangiectasia Mutated
**SLC7A11**	Catalytic subunit solute carrier family 7 member 11
**IREB-2**	Iron-responsive element-binding protein-2
**HO-1**	Heme oxygenase-1
**NCOA4**	Nuclear receptor coactivator 4
**Nrf2**	Nuclear factor erythroid 2-related factor 2
**PUFAs**	Polyunsaturated Fatty Acids
**ACSL4**	Acyl-CoA synthetase long-chain family member-4
**LPCAT-3**	Lysophosphatidylcholine acyltransferase-3
**PE**	Phosphatidylethanolamine
**LOX**	Lipoxygenases
**FAR1**	Fatty acyl-CoA reductase 1
**GNPAT**	Fatty acyl-CoA reductase 1 Glyceronephosphate O-acyltransferase
**mPTP**	Mitochondrial permeability transition pore
**SLC3A2**	Solute carrier family 3 member 2
**System Xc-**	Cystine/glutamate antiporter system
**TFAP2c**	Transcription Factor AP-2 Gamma
**Sp1**	Specificity Protein 1
**MVA**	Mevalonate
**IPP**	Isopentenyl pyrophosphate
**FSP1**	Ferroptosis suppressor protein 1
**AIFM2**	Apoptosis-inducing factor mitochondrial 2
**RTA**	Radical-trapping antioxidant
**CoQ10**	Coenzyme Q10
**GCH1**	GTP cyclohydrolase-1
**BH4/BH2**	Tetrahydrobiopterin/dihydrobiopterin
**DHODH**	dihydroorotate dehydrogenase
**DHO**	Dihydroorotate
**OA**	Orotoate
**mTOR**	Mammalian target of rapamycin
**GSDMD**	Gasdermin D
**PCI**	Percutaneous Coronary Intervention
**CABG**	Coronary artery bypass grafting
**LncRNA**	Long Non-Coding RNA
**MiRNA**	MicroRNA
**circHMGA2**	Circular RNA High Mobility Group AT-Hook 2
**NLRP3**	NOD-Like Receptor Pyrin Domain Containing 3
**LVEF**	Left ventricular ejection fraction
**CAD**	Coronary artery disease
**ELAVL1**	Embryonic lethal-abnormal vision like protein 1
**FOXC1**	Forkhead box C-1
**UPS**	Ubiquitin-proteasome system
**NEDD4L**	Neural Precursor Cell Expressed Developmentally Downregulated 4-Like
**YAP**	Yes-Associated Protein
**ERS**	Endoplasmic Reticulum Stress
**UPR**	Unfolded Protein Response
**ATF4-C**	Activating Transcription Factor 4 C-terminal domain
**EBP**	Emopamil-Binding Protein
**Chop**	C/EBP Homologous Protein
**NF-κB**	Nuclear Factor Kappa-Light-Chain-Enhancer of Activated B Cells
**JNK**	c-Jun N-terminal Kinase
**Pum2**	Pumilio 2
**PRDX6**	Peroxiredoxin 6
